# Thyroid function spectrum in Cushing’s syndrome

**DOI:** 10.1186/s12902-024-01614-4

**Published:** 2024-06-06

**Authors:** Peng Yu, Haoyue Yuan, Hong Chen, Xiaomu Li

**Affiliations:** 1 Department of Endocrinology, Zhongshan Hospital, Shanghai Medical College of Fudan University, Fenglin Road 180, Xuhui District, Shanghai, 200032 China; 2 Department of Endocrinology, Shanghai Geriatric Medical Center, Shanghai, 201104 China

**Keywords:** Cushing’s syndrome, Thyroid dysfunction, Diagnosis, Endocrine pathophysiology

## Abstract

**Purpose:**

Thyroid disorders have been reported in hypercortisolism patients. Endogenous Cushing’s syndrome (CS) potentially complicates its metabolic sequelae. We investigated thyroid function in CS patients to determine this relationship.

**Methods:**

In this cross-sectional study, we screened CS patients from 2016 to 2019 at our hospital. Patient demographic, medical history, and laboratory data were collected. Additionally, we performed a meta-analysis to demonstrate the prevalence of thyroid dysfunction in patients with CS.

**Results:**

Among 129 CS patients, 48.6% had triiodothyronine (TT3), 27.9% had thyroxine (TT4), 24.6% had free T3 (FT3), 27.7% had free T4 (FT4), and 6.2% had thyroid-stimulating hormone (TSH) levels below the reference values. Those with clinical CS showed more pronounced thyroid suppression than did those with subclinical CS. Cortisol levels were markedly greater in patients with pituitary hypothyroidism (*P* < 0.001). Serum cortisol levels throughout the day and post low-dose dexamethasone-suppression test (LDDST) results correlated with thyroid hormone levels, particularly in ACTH-independent CS. Correlations varied by thyroid status; FT3 and TSH were linked to cortisol in euthyroid individuals but not in those with low T3 or central hypothyroidism. TSH levels notably halved from the lowest to highest cortisol tertile post-LDDST. Finally, meta-analysis showed 22.7% (95% CI 12.6%-32.9%) central hypothyroidism in 528 CS patients of nine studies.

**Conclusion:**

Thyroid hormone levels are significantly correlated with cortisol levels and are impaired in patients with CS. However, the physiological adaptation and pathological conditions need further study.

## Introduction

Cushing’s syndrome (CS) is a multisystem metabolic disorder caused by the excessive secretion of cortisol. Accurate functional diagnosis determines the prognosis of the disease [1, 2]. Excessive glucocorticoids cause systemic organ damage and inhibit the function of the pituitary-–adrenal axis, interfering with the adenohypophysis [3, 4]. Glucocorticoids also affect pituitary–-thyroid axis function [5], but the dysfunction or clinical implications of thyroid function in patients with CS have not been determined.

To understand the effect of hypercortisolism on thyroid function, Shekhar et al. [5] performed a cohort study and reported that abnormal thyroid function, likely mediated by subnormal nocturnal thyroid-stimulating hormone (TSH), is prevalent in patients with CS and is reversible after curative treatment. Although another report revealed that serum TSH and free thyroxine (FT4) levels were decreased in patients with CS [1], many CS patients have high or normal TSH levels and low free triiodothyronine (FT3) levels [6]. Elucidating thyroid function in CS patients may deepen our understanding of crosstalk between the anterior pituitary gland and CS patients.

## Methods

### Study population

A retrospective study of CS patients treated at the Department of Endocrinology, Zhongshan Hospital, from 2016 to 2019 was conducted. All the participants met the diagnostic criteria of CS based on the Endocrine Society guidelines [2]. According to whether the patient was complicated with specific signs or symptoms of Cushing’s syndrome, the CS was categorized as clinical or subclinical [7].

Inclusion criteria: Elevated late-night serum cortisol (≥ 7.5 µg/dl) or serum cortisol ≥ 1.8 µg/dL after an overnight 1 mg dexamethasone-suppression test (DST). Exclusion criteria: (1) TPO-Ab, TG-Ab or TR-Ab positivity; (2) abnormal liver or renal function; (3) Exogenous glucocorticoid administration; (4) Seronegative primary hypothyroidism; and (5) hyperthyroidism defined as raised FT4/FT3 and suppressed TSH. Finally, a total of 129 patients were enrolled for the analysis. All procedures complied with the Helsinki Declaration and were approved by the Zhongshan Hospital Ethics Committee, Fudan University, China.

### General clinical data collection and definition

Demographic and lifestyle data were extracted from electronic medical records (EMRs) of our hospital, including age, sex, and medication history. According to the clinical routine protocol, standardized measurements of body weight and height were performed on every subject and recorded in their clinical history. After overnight fasting for 12 h, blood samples were collected for thyroid hormone and biochemical parameter detection (Japan Hitachi 7600 biochemical analyser), usually in the next morning of admission. When the cortisol rhythm test had an abnormal result, we routinely performed the LDDST after the third day of admission.

The reference ranges quoted by the manufacturer for TSH are 0.27–4.2 µIU/mL, for FT4, 12.0–22.0 pmol/L, for TT4, 66.0-181.0 nmol/L, for TT3, 1.3–3.1 nmol/L and for FT3, 2.8–7.1 pmol/L.

Low-T3 syndrome is defined as a low level of TT3 and normal FT4. Central hypothyroidism is defined as a laboratory test showing low or normal serum TSH levels in the presence of low FT4 levels.

Subclinical hypothyroidism is characterized by an elevated serum TSH level of 4.2–10µIU/mL, while the serum FT4 remains within the normal range.

### Meta-analysis

We performed this analysis as previously reported [8]. Study selection: A systematic literature search was performed in PubMed and the Cochrane Library according to the Meta-analyses of Observational Studies in Epidemiology (MOOSE) guidelines [9]. The terms “Cushing’s syndrome,” “Cushing syndrome,” “Cushing’s disease,” “Cushing disease,” “pituitary ACTH hypersecretion” and “Thyroid” in the title or abstract were used to identify published articles reporting on Cushing’s syndrome/disease. The literature search was performed in March 2022.

Data extraction: Abstracts were screened by one of the authors to identify clinical studies. Then, the full-text manuscript of each eligible study was screened for content reporting on the ratio or value of thyroid dysfunction in the sample or cohort. To obtain data on the prevalence of central hypothyroidism in CS for all studies, we contacted the corresponding authors of the studies with missing data.

### Statistical analysis

Continuous variables with a normal distribution are expressed as mean ± standard deviation. Nonnormal variables are expressed as median with interquartile range. Analysis of variance (ANOVA) and Student’s *t*-test were used to determine differences in means between groups. Partial Spearman’s correlation analysis was performed to identify correlations. The effect of serum cortisol on thyroid hormones was further evaluated by dividing cortisol concentrations into tertiles. STATA 18.0 for windows (Stata-Corp, College Station, TX, USA) was used. All statistical tests were two-tailed, and *p* < 0.05 was regarded as statistically significant.

## Results

### Thyroid function state and general characteristics and of the CS patients

**Fig. 1:**
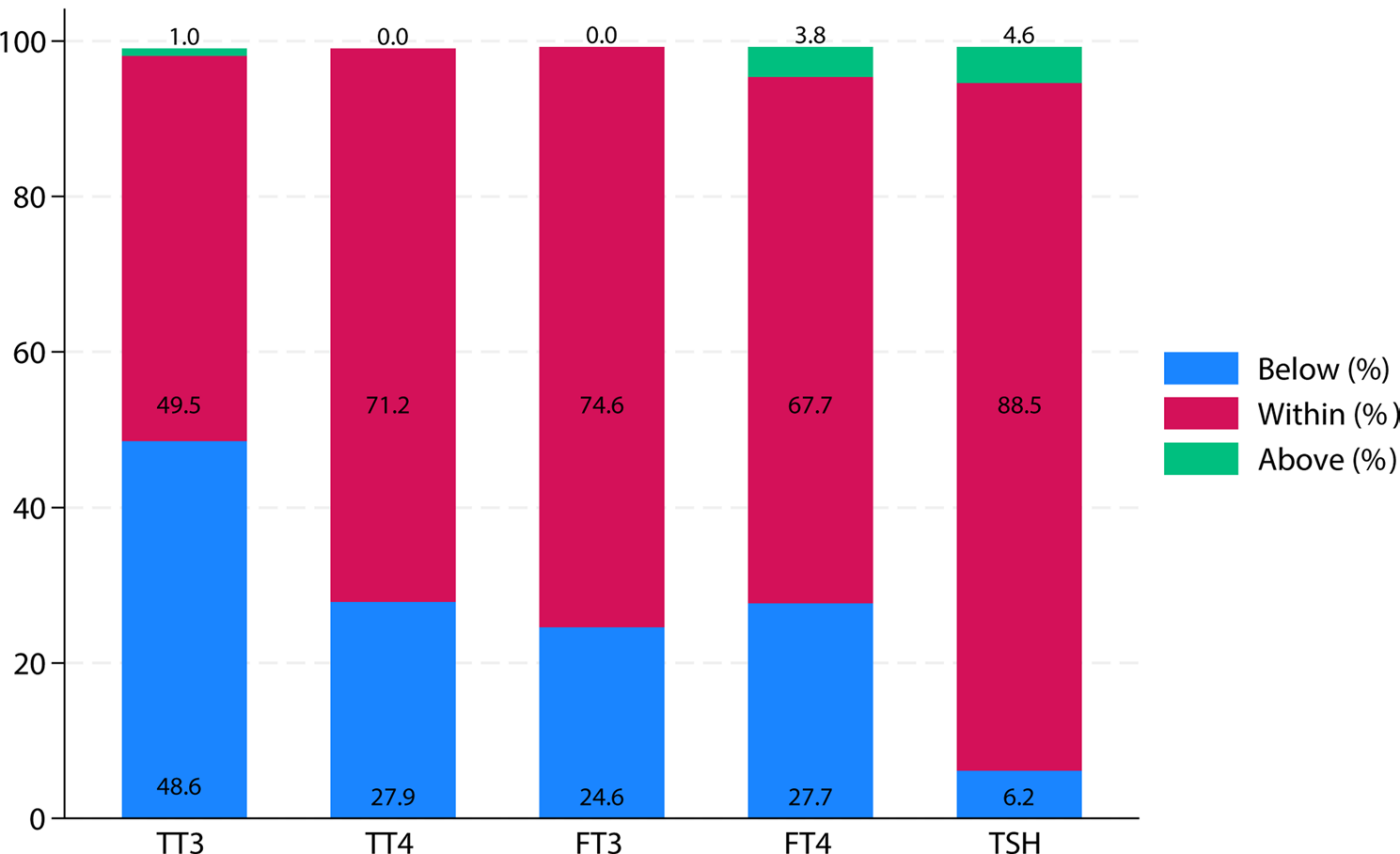
Thyroid hormone distribution in the study population

We classified the thyroid functional into different categories according to the laboratory reference ranges, founding TT3, TT4, FT3, FT4 and TSH levels were below the reference range in 48.6%, 27.9%, 24.6%, 27.7% and 6.2% of 129 CS patients, respectively (Fig. 1).

The general characteristics of the participants are presented in Table 1. Among the 129 patients with CS, 28 (21.7%) patients were male, with an average age of 50.08 ± 14.00 years and a mean BMI of 25.20 ± 3.88 years. The CS patients were further divided into clinical and subclinical groups according to the presence or absence of symptoms. There were 29 subclinical CS patients (26 females and 3 males; mean age 55.84 ± 12.07 years) and 100 clinical CS patients (75 females and 25 males; mean age 48.45 ± 14.14 years). The patients with clinical CS were younger and had higher serum cortisol levels at 0800, 1600 and 2400 and lower potassium and lower thyroid hormone levels, except for TT4, than those in the subclinical patients (*p* < 0.05). There were more ACTH-dependent tumours and central hypothyroidism in the clinical CS group than in the subclinical CS group, indicating more pituitary and ectopic CS in clinical CS group, while surgical treatment and adenoma size were comparable in both groups according to location. Sex, BMI, serum sodium concentration and TT4 level were similar between the two groups (*p* > 0.05) (Table 1).

**Table 1:** General characteristics of patients with Cushing’s syndrome

Variables	Clinical (*n* = 100)	Subclinical (*n* = 29)	All (*n* = 129)	*p*-Value
Age (years)	48.45 ± 14.14	55.84 ± 12.07	50.08 ± 14.00	0.019
Male, %	25 (25.0%)	3 (10.3%)	28 (21.7%)	0.092
BMI, kg/m^2^	25.11 ± 4.07	25.58 ± 3.12	25.20 ± 3.88	0.61
Sodium, mmol/L	143.77 ± 3.57	142.58 ± 2.35	143.50 ± 3.36	0.096
Potassium, mmol/L	3.59 ± 0.72	3.95 ± 0.51	3.67 ± 0.69	0.012
Cortisol 0800 (nmol/L)	668.17 ± 335.29	399.06 ± 135.10	606.72 ± 321.65	< 0.001
Cortisol 1600 (nmol/L)	594.13 ± 314.92	309.80 ± 173.36	527.63 ± 311.83	< 0.001
Cortisol 2400 (nmol/L)	510.55 ± 269.71	218.80 ± 107.88	442.32 ± 271.32	< 0.001
Cortisol after LDDST (nmol/L)	509.25 ± 276.75	252.16 ± 157.07	447.36 ± 275.57	< 0.001
T3 (nmol/L)	1.20 ± 0.31	1.55 ± 0.73	1.27 ± 0.46	< 0.001
T4 (nmol/L)	79.10 ± 22.80	87.53 ± 20.94	80.90 ± 22.59	0.121
FT3 (pmol/L)	3.27 ± 0.88	3.93 ± 0.78	3.42 ± 0.90	< 0.001
FT4 (pmol/L)	13.95 ± 3.37	15.57 ± 3.25	14.32 ± 3.40	0.024
TSH (µIU/mL)	1.15 ± 0.99	2.25 ± 1.76	1.40 ± 1.29	< 0.001
Thyroid status				0.005
Euthyroid	42 (42.0%)	19 (65.5%)	61 (47.3%)	
Pituitary hypothyroidism	33 (33.0%)	1 (3.4%)	34 (26.4%)	
Low-T3	22 (22.0%)	6 (20.7%)	28 (21.7%)	
Subclinical hypothyroidism	3 (3.0%)	3 (10.3%)	6 (4.7%)	
ACTH-dependent	39 (39.0%)	1 (3.4%)	40 (31.0%)	< 0.001
Location				< 0.001
Adrenal	61 (61.0%)	28 (96.6%)	89 (69.0%)	
Adenoma	57 (93.4%)	28 (100.0%)	85 (95.5%)	
Carcinoma	4 (6.6%)	0 (0.0%)	4 (4.5%)	
Size, cm	2.93 ± 1.97	2.71 ± 0.87	2.86 ± 1.70	
Surgery	53 (86.9%)	21 (75.0%)	74 (83.1%)	
Pituitary	32 (32.0%)	0 (0.0%)	32 (24.8%)	
Size, mm	0.42 ± 0.40	0		
Surgery	18 (56.2%)	0		
Ectopic	7 (7.0%)	1 (3.4%)	8 (6.2%)	

BMI, body mass index. LDDST, low-dose dexamethasone-suppression test

### General characteristics and cortisol levels of patients with different thyroid function subtypes

To elucidate the thyroid disorders of the CS, we divided the patients into three groups according to thyroid functional phenotype, and analysed demographic characteristics and cortisol level. The 129 patients with CS were divided into euthyroid group (61 patients, 18 males; mean age 52.60 ± 14.03 years), low-T3 group (28 patients, 3 males; mean age 46.53 ± 14.34 years) and central hypothyroidism group (34 patients, 6 males; mean age 46.03 ± 11.47 years) according to their thyroid hormone levels. We investigated the clinical parameters between the three groups (Table 2). Age, gender and BMI were similar among the three group (*p* > 0.05). The patients with central hypothyroidism had the lowest thyroid hormones and highest serum cortisol levels at 0800, 1600, 2400 and after LDDST (*p* < 0.001 vs. both other groups) (Table 2). However, we did not measure thyroid function after the LDDST, but previous reports have indicated that this change can be suppressed by dexamethasone [10].

**Table 2:** General characteristics of patients with or without hypothyroidism

		Assessment of thyroid function		
Variables	Euthyroid (*n* = 61)	Low T3 (*n* = 28)	Central hypothyroidism (*n* = 34)	*p*-value
Age (years)	52.60 ± 14.03	46.53 ± 14.34	46.03 ± 11.47	0.055
Sex (M/F)	18 (29.5%)	3 (10.7%)	6 (17.6%)	0.107
BMI (kg/m^2^)	25.42 ± 3.49	24.05 ± 4.47	25.47 ± 3.60	0.285
Sodium (mmol/L)	142.83 ± 3.38	143.71 ± 2.66	144.55 ± 3.73	0.055
Potassium (mmol/L)	3.76 ± 0.59	3.72 ± 0.55	3.45 ± 0.90	0.099
T3 (nmol/L)	1.60 ± 0.49	1.02 ± 0.13	0.98 ± 0.25	< 0.001
T4 (nmol/L)	100.25 ± 18.87	72.30 ± 8.64	59.76 ± 11.89	< 0.001
FT3 (pmol/L)	4.00 ± 0.73	3.05 ± 0.53	2.64 ± 0.65	< 0.001
FT4 (pmol/L)	16.73 ± 2.89	13.77 ± 1.25	10.54 ± 1.20	< 0.001
TSH (µIU/mL)	1.50 ± 0.97	0.92 ± 0.66	0.85 ± 0.68	< 0.001
Cortisol 0800 (nmol/L)	464.47 ± 205.78	667.53 ± 296.60	825.80 ± 345.37	< 0.001
Cortisol 1600 (nmol/L)	396.26 ± 228.83	585.64 ± 261.00	739.42 ± 298.92	< 0.001
Cortisol 2400 (nmol/L)	316.90 ± 208.15	517.01 ± 228.87	651.03 ± 275.39	< 0.001
Cortisol after LDDST	329.63 ± 235.51	545.81 ± 221.29	628.15 ± 261.08	< 0.001

BMI, body mass index. LDDST, low-dose dexamethasone-suppression test

### Correlations between serum cortisol and thyroid hormones in CS patients

**Fig. 2:**
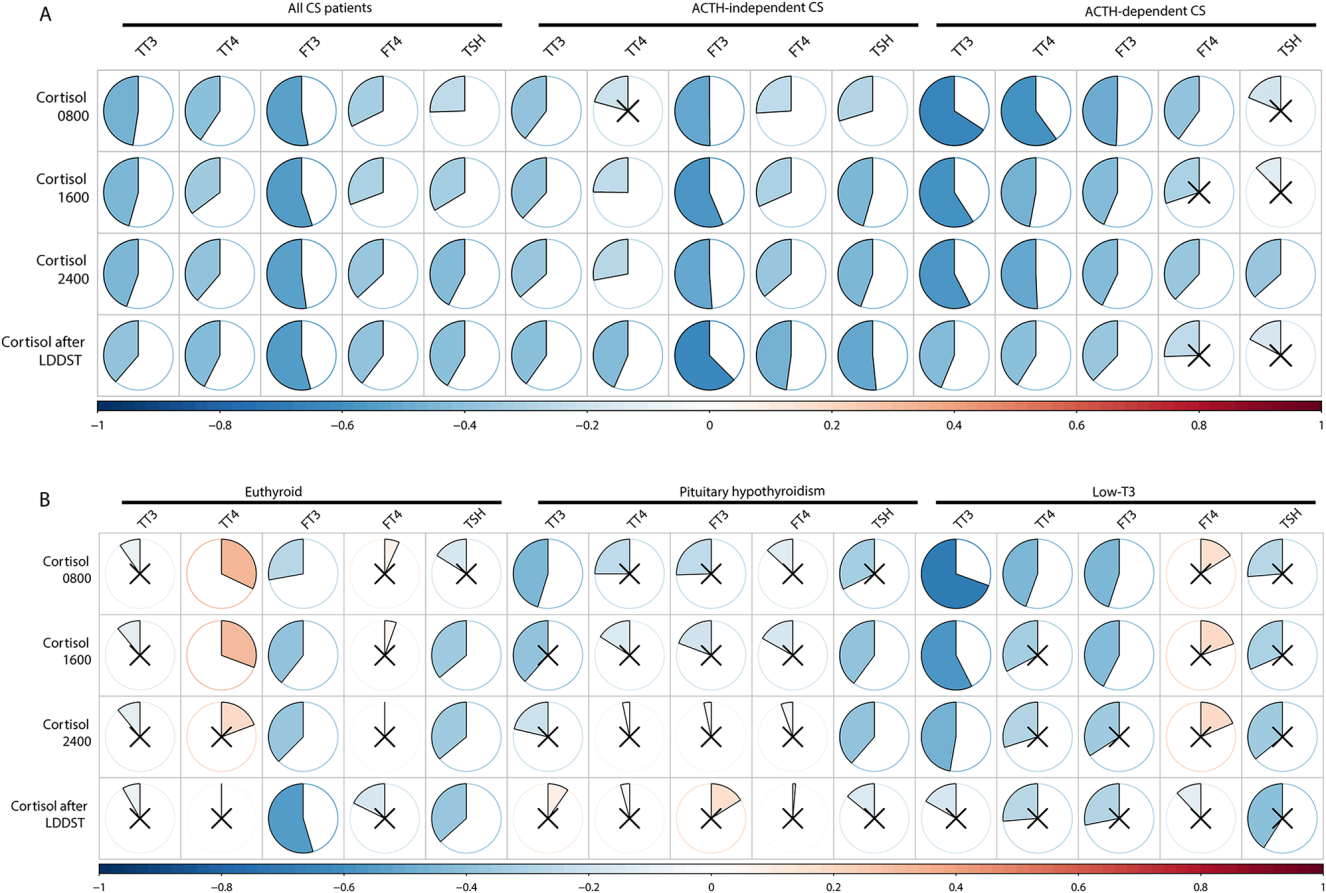
Correlations between serum cortisol and thyroid hormone levels. (**A**) All patients and ACTH-dependent or ACTH-independent individuals; (**B**) Different thyroid function groups

Because the analysis according to thyroid function showed elevated serum cortisol levels with the exacerbation of thyroid dysfunction, we assessed the correlations between the levels of serum cortisol and thyroid-related hormones in all patients (Fig. 2A). Significant negative correlations were detected between serum cortisol levels at 0800, 1600, 2400 or after the LDDST and thyroid-related parameters, including T3, T4 FT3, FT4, and TSH. This correlation was less evident in the ACTH-dependent CS and was further weakened in the low-T3 subgroup and central hypothyroidism subgroup (Fig. 2B).

### Autonomous secretion of cortisol suppresses thyroid hormones

The serum cortisol level after LDDST, which reflects the autonomous and pathological secretion in the CS [11], could show the distinct effect of excessive cortisol levels on thyroid function. Additionally, we stratified the patients by tertile according to serum cortisol levels after LDDST into a low-tertile group (Tertile 1), medium-tertile group (Tertile 2) and high-tertile group (Tertile 3) separately to assess the dose–response relationship (Table 3). There were no differences in sex, age or BMI among the three groups. All of the thyroid hormones decreased significantly with increasing serum cortisol after the LDDST. The thyroid hormone levels in Tertile 3 were the lowest of all the groups, with TSH reduced by more than half, indicating central suppression by excessive cortisol (Table 3). All carcinomas were in Tertile 3, and adenomas were larger in the higher cortisol tertile than in the lower cortisol tertile.

**Table 3:** General characteristic of patients according to serum cortisol level tertiles after LDDST

Variables		Cortisol after LDDST		*p*-value
	Tertile 1 (< 284)	Tertile 2 (284-564.2)	Tertile 3 (> 564.2)	
Cortisol after LDDST (nmol/L)	158.19 ± 55.60	422.12 ± 88.22	761.78 ± 181.47	< 0.001
Age (years)	54.46 ± 15.42	48.39 ± 11.23	47.26 ± 12.54	0.071
Sex (M/F)	7 (19.4%)	7 (19.4%)	10 (27.8%)	0.617
BMI (kg/m^2^)	25.78 ± 3.99	24.55 ± 3.45	24.93 ± 3.65	0.425
T3 (nmol/L)	1.57 ± 0.63	1.19 ± 0.29	1.07 ± 0.27	< 0.001
T4 (nmol/L)	94.53 ± 23.43	76.48 ± 18.96	70.28 ± 18.07	< 0.001
FT3 (pmol/L)	4.03 ± 0.74	3.38 ± 0.82	2.84 ± 0.61	< 0.001
FT4 (pmol/L)	16.11 ± 3.49	14.29 ± 3.33	12.79 ± 2.87	< 0.001
TSH (µIU/mL)	2.25 ± 1.65	1.22 ± 0.78	0.81 ± 0.75	< 0.001
ACTH-independent	25 (69.4%)	26 (72.2%)	24 (66.7%)	0.877
Carcinoma	0 (0.0%)	0 (0.0%)	4 (16.7%)	0.011
Adenoma	25 (100.0%)	26 (100.0%)	20 (83.3%)	
Size, cm	2.38 ± 0.97	2.59 ± 0.47	2.81 ± 1.78	0.076

### Thyroid dysfunction prevalence rate in CS

**Fig. 3:**
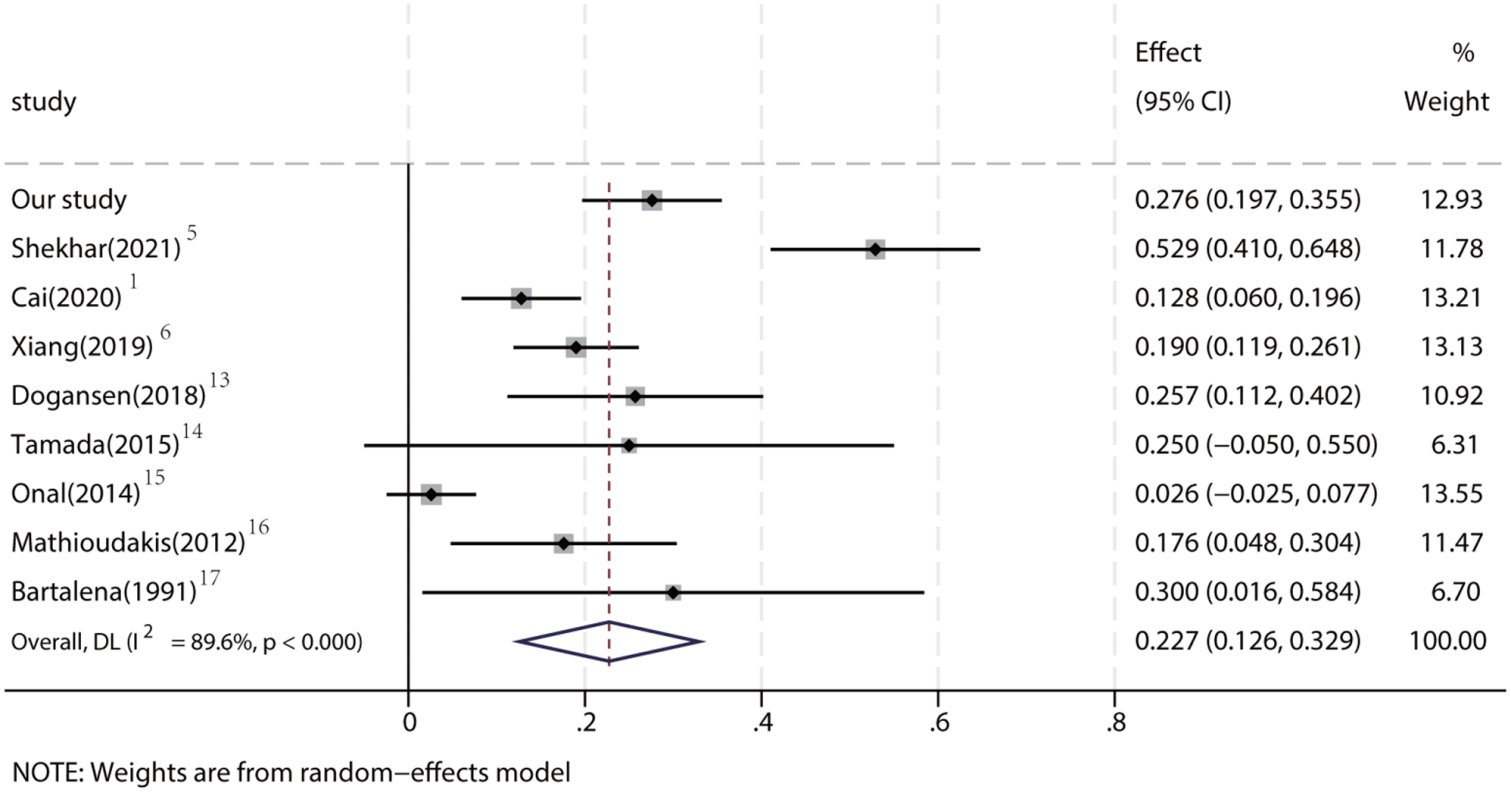
Forest plot of central hypothyroidism dysfunction prevalence in CS

Our data and those of others showed that hypercortisolism can exert important effects on the pituitary–thyroid axis, leading to thyroid dysfunction [12]. In CS patients, excessive cortisol secretion causes impaired hypothalamus–pituitary–thyroid axis activity. We performed a literature review and meta-analysis of the incidence of central thyroid dysfunction in patients with CS. The analysis included nine studies, with a total of 528 enrolled patients with CS. These patients had a central hypothyroidism rate of 22.7% (95% CI 12.6–32.9%) (Fig. 3).

## Discussion

Thyroid dysfunction is common in CS patients [18], but the prevalence of thyroid dysfunction in CS patients has not been investigated in depth. Our study focused on the spectrum of thyroid function in CS patients, revealing a notable incidence of thyroid hormone alterations, including decreased levels of triiodothyronine (TT3), thyroxine (TT4), free T3 (FT3), free T4 (FT4), and thyroid-stimulating hormone (TSH) below the reference values in a substantial proportion of our cohort. These findings are consistent with the known effects of glucocorticoid excess on the hypothalamic-pituitary-thyroid (HPT) axis, which can lead to various degrees of central hypothyroidism or non-thyroidal illness syndrome (NTIS), characterized by alterations in thyroid hormone levels without overt hypothyroid symptoms.

The prevalence of thyroid hormone changes in CS patients is an area of active investigation, with implications for diagnosis, management, and prognosis. Previous studies have reported varied prevalence rates of thyroid dysfunction in CS, reflecting the heterogeneity of the studied populations, the criteria used to define thyroid abnormalities, and the severity and duration of cortisol excess. Our meta-analysis, including nine studies with a total of 528 CS patients, demonstrated a central hypothyroidism prevalence of approximately 22.7%. This aligns with findings from other cohorts and underscores the importance of systematic screening for thyroid function abnormalities in CS patients.

The prevalence of thyroid hormone changes in CS patients is an area of active investigation, with implications for diagnosis, management, and prognosis. Previous studies have reported varied prevalence rates of thyroid dysfunction in CS, reflecting the heterogeneity of the studied populations, the criteria used to define thyroid abnormalities, and the severity and duration of cortisol excess. Our meta-analysis, including nine studies with a total of 528 CS patients, demonstrated a central hypothyroidism prevalence of approximately 22.7%. This aligns with findings from other cohorts and underscores the importance of systematic screening for thyroid function abnormalities in CS patients.

To determine whether the clinical manifestations of CS are influenced by cortisol or thyroid hormones, we compared clinical and subclinical CS and found a significant difference only in the serum cortisol levels, which disappeared after the LDDST. In the clinical groups, levels of thyroid hormones, except for TT4, were significantly reduced, with a greater incidence of central hypothyroidism.

We further divided the CS patients into three groups according to their thyroid function status: euthyroid, low T3 and central hypothyroidism. The central hypothyroidism group exhibited significant pituitary–thyroid axis suppression, accompanied by significant elevation of serum cortisol, which was not suppressed by the LDDST.

During illness, T4 to T3 is downregulated. This is called “low T3 syndrome”, an adaptive metabolic mechanism to reduce energy expenditure and prevent catabolism, recently reported very prevalent in medical inpatients outside the critically ill setting [19]. In cohort of patients at nutritional risk, the prevalence of low T3 syndrome was 61% [19]. Our study showed 21% complicated low T3 syndrome in the CS patients, indicating the prevention of catabolism.

The mechanisms underlying thyroid hormone changes in CS are multifaceted, involving direct and indirect effects of cortisol on thyroid hormone synthesis, metabolism, and action. Cortisol may suppress TSH secretion at the pituitary level, alter the peripheral conversion of T4 to T3, and modulate thyroid hormone action at the cellular level through effects on thyroid hormone receptors and transporters. Additionally, the role of glucocorticoids in modulating immune responses may have implications for thyroid autoimmunity in CS.

Moreover, CS can increase visceral fat accumulation [20]. Adipose tissue releases cortisol, increases local glucocorticoid signalling and contributes to cortisol regeneration [21]. To exclude nonautonomous cortisol interference, we analysed the correlations after the LDDST, and all thyroid hormones were negatively correlated with serum cortisol, with more pronounced correlations than the basal levels.

The correlation between excess glucocorticoids and suppressed TSH has been extensively studied in animal studies. The precise mechanisms involved in the depressed pituitary–thyroid axis in patients with CS have not been studied in detail. In humans, hypercortisolism can reduce TSH secretion and TSH pulses. TSH levels are lower in CS patients than in controls [22]. The physiologic nocturnal increase in TSH also decreases due to the decrease in the nocturnal TSH surge [23], which was confirmed in a recent retrospective cohort study [5]. An inverse relationship between night serum cortisol and TSH in CS patients has been reported.

Hypercortisolism can be associated with biochemical features characteristic of central hypothyroidism; reduced T4, T3, and FT3; and increased reverse T3 levels, which have various probable mechanisms [18].

The hypothalamic–pituitary–adrenal and hypothalamic–pituitary–thyroid axes are closely linked [24]. Most importantly, cortisol acts directly on TRH secretion, possibly via the glucocorticoid element response to DNA in TRH neurons [25]. Some studies suggest that excessive cortisol may induce a decrease in TRH mRNA levels in the midcaudal paraventricular nucleus (PVN) [25]. Regardless of the underlying mechanism, excessive cortisol levels could account for the blunted HPT responses in the CS.

This study has several limitations. The study had a cross-sectional design and lacked follow-up data. Therefore, a causal relationship between hypercortisolism and thyroid dysfunction cannot be determined. The sample size was not estimated before the study and was small. Furthermore, there is a lack of mechanistic insight into the potential pathophysiological or physiologic adaptive role of thyroid dysfunction in CS.

In summary, cortisol and thyroid hormone levels are correlated in CS patients, especially after the LDDST. A meta-analysis of previous reports demonstrated that the prevalence of thyroid dysfunction is approximately 22% in CS patients. However, additional biochemical studies are needed to elucidate the physiological adaptation or pathological conditions underlying the association between CS and thyroid dysfunction.

## Data Availability

The data that support the findings of this study are available from the corresponding author upon reasonable request.
